# Periodic Fluctuation of Tidal Volumes Further Improves Variable Ventilation in Experimental Acute Respiratory Distress Syndrome

**DOI:** 10.3389/fphys.2018.00905

**Published:** 2018-07-12

**Authors:** Andreas Güldner, Robert Huhle, Alessandro Beda, Thomas Kiss, Thomas Bluth, Ines Rentzsch, Sarah Kerber, Nadja C. Carvalho, Michael Kasper, Paolo Pelosi, Marcelo G. de Abreu

**Affiliations:** ^1^Department of Anesthesiology and Intensive Care Medicine, Pulmonary Engineering Group, University Hospital Carl Gustav Carus, Technische Universität Dresden, Dresden, Germany; ^2^Departamento de Engenharia Eletrônica, Federal University of Minas Gerais, Belo Horizonte, Brazil; ^3^Department of Orthodontics, Technische Universität Dresden, Dresden, Germany; ^4^Institute of Anatomy, Technische Universität Dresden, Dresden, Germany; ^5^Department of Surgical Sciences and Integrated Diagnostics, IRCCS San Martino IST, University of Genoa, Genoa, Italy

**Keywords:** experimental model, acute respiratory distress syndrome, mechanical ventilation, variable ventilation, gas exchange, respiratory mechanics, lung inflammation, lung damage

## Abstract

In experimental acute respiratory distress syndrome (ARDS), random variation of tidal volumes (*V*_*T*_) during volume controlled ventilation improves gas exchange and respiratory system mechanics (so-called stochastic resonance hypothesis). It is unknown whether those positive effects may be further enhanced by periodic *V*_*T*_ fluctuation at distinct frequencies, also known as deterministic frequency resonance. We hypothesized that the positive effects of variable ventilation on lung function may be further amplified by periodic *V*_*T*_ fluctuation at specific frequencies. In anesthetized and mechanically ventilated pigs, severe ARDS was induced by saline lung lavage and injurious *V*_*T*_ (double-hit model). Animals were then randomly assigned to 6 h of protective ventilation with one of four *V*_*T*_ patterns: (1) random variation of *V*_*T*_ (WN); (2) P_04_, main *V*_*T*_ frequency of 0.13 Hz; (3) P_10_, main *V*_*T*_ frequency of 0.05 Hz; (4) VCV, conventional non-variable volume controlled ventilation. In groups with variable *V*_*T*_, the coefficient of variation was identical (30%). We assessed lung mechanics and gas exchange, and determined lung histology and inflammation. Compared to VCV, WN, P_04_, and P_10_ resulted in lower respiratory system elastance (63 ± 13 cm H_2_O/L vs. 50 ± 14 cm H_2_O/L, 48.4 ± 21 cm H_2_O/L, and 45.1 ± 5.9 cm H_2_O/L respectively, *P* < 0.05 all), but only P_10_ improved PaO_2_/F_I_O_2_ after 6 h of ventilation (318 ± 96 vs. 445 ± 110 mm Hg, *P* < 0.05). Cycle-by-cycle analysis of lung mechanics suggested intertidal recruitment/de-recruitment in P_10_. Lung histologic damage and inflammation did not differ among groups. In this experimental model of severe ARDS, periodic *V*_*T*_ fluctuation at a frequency of 0.05 Hz improved oxygenation during variable ventilation, suggesting that deterministic resonance adds further benefit to variable ventilation.

## Introduction

Mechanical ventilation with variable tidal volumes (*V*_*T*_) has been shown to improve gas exchange as well as lung mechanics (Lefevre et al., 1996; Suki et al., 1998; Mutch et al., 2000, 2005; Funk et al., 2004; Bellardine et al., 2006; Graham et al., 2011a,b) and attenuate ventilator induced lung injury (VILI) (Boker et al., 2002; Spieth et al., 2009b; Thammanomai et al., 2013; de Magalhães et al., 2016; Samary et al., 2016) in different models of the acute respiratory distress syndrome (ARDS). In these studies, artificial as well as biologically variable *V*_*T*_ patterns have been investigated.

During variable ventilation, *V*_*T*_ patterns are often described using the coefficient of variation (CV), even when probability density distributions are not strictly normal. Early investigations focused on the optimization of CV to improve lung function (Arold et al., 2002; Spieth et al., 2009a) and attenuate lung injury (Kiss et al., 2016). In oleic acid induced lung injury in porcine, a CV ≥ 40% was associated with an optimal combination of gas exchange and lung mechanics, while a CV of 30% attenuated VILI (Kiss et al., 2016).

More recently, different *V*_*T*_ probability density distributions were investigated in mice. In lung injury induced by hydrochloric acid, a *V*_*T*_ probability density distribution tailored to maximize recruitment allowed better gas exchange and respiratory mechanics with reduced lung inflammation compared to random variable ventilation (Thammanomai et al., 2008, 2013).

Aside from this, advanced *V*_*T*_ pattern properties (e.g., power distribution, auto-correlation, complexity) have been only addressed once in a model of oleic acid induced lung injury in pigs comparing biologically and random white noise variable *V*_*T*_ patterns (Froehlich et al., 2008). Both patterns yielded similar improvements in gas exchange and lung mechanics arguably because both patterns did not differ relevantly in terms of auto-correlation, given the possible range of values. Except for a study in a numerical model of respiratory mechanics (Ma et al., 2011) the impact of determinism during randomly variable ventilation have not been addressed in experimental models of ARDS yet.

In the present study, we investigated the effects of distinct *V*_*T*_ pattern frequencies, so-called deterministic frequency resonance, on gas exchange, lung mechanics, hemodynamics, histology, and inflammation during variable controlled mechanical ventilation in experimental ARDS. We hypothesized that, during volume controlled mechanical ventilation, deterministic frequency resonance through *V*_*T*_ fluctuation improves lung function and reduces lung injury during variable ventilation.

## Methods

The local animal care committee approved the experimental protocol (TVA 24-9168.11-1/2011-22) (Landesdirektion Dresden, Dresden, Saxony, Germany).

### Anesthesia and initial ventilator settings

In total, 40 female pigs with mean body weight of 40.4 kg (29.9–50.5 kg, German landrace) were investigated. Animals were pre-medicated intramuscularly with 10 mg/kg ketamine (Ketamin-ratiopharm; Ratiopharm, Ulm, Germany) and 1 mg/kg midazolam (Midazolam, Ratiopharm, Ulm, Germany), and had the trachea intubated with a cuffed 8.0-mm ID endotracheal tube. Initially, mechanical ventilation (Evita XL, Dräger Medical, Lübeck, Germany) was performed in volume-controlled mode with the following settings: fraction of inspired oxygen (FIO_2_) = 1.0, *V*_*T*_ = 10 mL/kg, positive end-expiratory pressure (PEEP) = 5 cm H_2_O, inspiratory to expiratory time ratio (I:E) = 1:1 and respiratory rate (RR) to keep PaCO_2_ in the range of 35–45 mm Hg.

Anesthesia was maintained by means of continuous intravenous infusion of midazolam (1–2 mg/kg/h) and ketamine (10–20 mg/kg/h). Muscle paralysis was achieved by continuous administration of atracurium (1–2 mg/kg/h), whereas the volume status was maintained with a continuous infusion of Ringer's acetate (RA- Ringer-Acetat-Lösung Bernburg, Serumwerk Bernburg AG, Bernburg, Germany) at 10 mL/kg/h. The external jugular vein and internal carotid artery were cannulated with 8.5 Fr. Sheaths. The arterial line was used for continuous blood pressure measurements and blood sampling. A pulmonary artery catheter (Opticath, Abbott, Abbott Park, Chicago, IL, USA) was advanced through the venous sheath into the pulmonary artery for continuous measurement of pulmonary arterial blood pressure, mixed venous blood sampling and cardiac output measurements. During the whole experiments, animals were kept supine.

### Induction of lung injury

Lung injury was induced by saline lung lavage and injurious mechanical ventilation (double-hit model), as described elsewhere (Silva et al., 2013). Briefly, multiple lavages with warmed normal saline solution was performed until PaO_2_/FIO_2_ fell below 200 mm Hg and remained stable at this level for ≥ 30 min (1st hit). Following that, ventilator induced lung injury was performed with the following settings: driving pressure of 60 cm H_2_O, PEEP = 0 cm H_2_O, RR = 10 min^−1^, for 5 min (2nd hit). After the 2nd hit previous ventilator settings at baseline 1 were resumed, resulting in PaO_2_/FIO_2_ < 100 mmHg. Lung injury was considered stable, when PaO_2_ did not increase within 15 min.

### Patterns of tidal volume variability

It was shown in the literature that the positive effects of variable ventilation with random variation of *V*_*T*_ depend on the probability distribution in general and more specifically on the coefficient of variation (stochastic resonance). Therefore, in order to delineate potential effects of deterministic frequency resonance and stochastic resonance, *V*_*T*_ patterns were constructed with the constraint of identical probability distribution (Suki et al., 1998; Brewster et al., 2005). Deterministic resonance requires a (sub-) system to be stimulated by frequency specific excitation. Potential sub-systems related directly to the respiratory system have dynamic characteristics (Table 1 and Supplementary Material).

**Table 1:** Dynamics of sub-systems and processes related to respiration.

**Sub-system/Process**	**τ in s**	**Frequency in Hz**	**References**
Lung Recruitment	1.8 … 10.7	0.09 … 0.60	Neumann et al., 1998; Markstaller et al., 2001
Ca2+ mobilization	9 … 13	0.08 … 0.11	Wirtz and Dobbs, 1990
Lung derecruitment	20 … 26.5	0.04 … 0.05	Haller et al., 1998; Majumdar et al., 2012
Surfactant prod./release	19.7 … 94.6	0.01 … 0.05	Bates and Irvin, 2002; Massa et al., 2008
HPV	120.151	0.008 … 0.007	Sylvester et al., 2012

*HPV, Hypoxic pulmonary vasoconstriction*.

**Figure 1:**
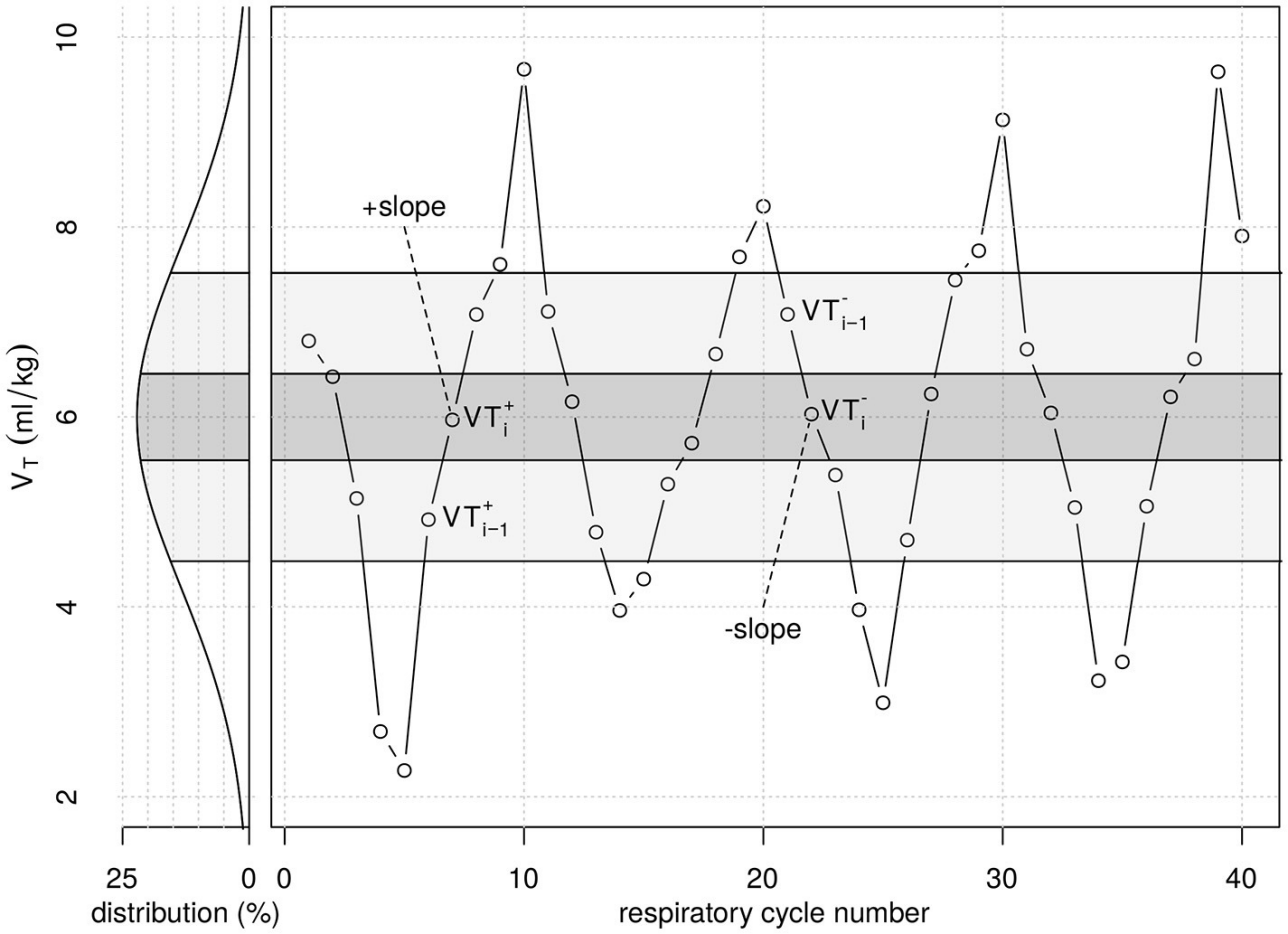
Graphical illustration of part of the tidal volume (*V*_*T*_) series from period pattern P_10_. Original *V*_*T*_ values were randomly generated following a Gaussian distribution (white noise), with respective probability density (*left*). *V*_*T*_ values were then taken from each of the five probability density regions shown (quintiles of the original random *V*_*T*_ series) and re-ordered from lowest to highest (+*slope*) and highest to lowest (−*slope*) *V*_*T*_ values, repetitively. *V*_*T*_i−1__ represents *V*_*T*_ in the second quintile during +*slope*, while *V*_*T*_i−1__ represents *V*_*T*_ in the fourth quintile during −*slope*. *V*_*T*_i+__ and V_Ti−_ represent *V*_*T*_ in the third quintile during−*slope* and +*slope*, respectively, which value is close to the mean of 6 mL/kg. The period pattern P_04_ was generated similarly, dividing the probability distribution curve into two density regions only.

Three sequences of *V*_*T*_ cycles with distinct variability patterns were constructed. Initially, a random white noise *V*_*T*_ sequence with Gaussian distribution was generated using Matlab version R14 (Natick, MA, USA) (WN). The probability density histogram of the original sequence was subdivided in two, respectively in five sections of equal areas under the Gaussian curve. In this way two *V*_*T*_ sequences were generated taking one value of each consecutive area under the curve, as shown in Figure 1. Each *V*_*T*_ pattern consisted of the exactly same 600 values and thus had identical probability density distribution (cmp. Supplementary Table 1). At a mean respiratory rate of 30 bpm each pattern had a length of ~20 min. Each pattern was repeated 18 times during the 6 h of therapy. Mean *V*_*T*_ of all patterns was 6 ml/kg and single cycle *V*_*T*_ values ranged from 0.6 to 11.4 ml/kg (99.7% of all values). 95% of all *V*_*T*_ values were in the range of 2.4 … 9.6 ml/kg.

Accordingly, the pattern of the second sequence showed periodic recurrence every four cycles (P_04_). Considering a respiratory frequency of 30 min^−1^ (the average value used in the present study), this periodicity corresponds to a main frequency of 0.13 Hz, coinciding with the dynamics of lung recruitment and stretch induced calcium mobilization (Wirtz and Dobbs, 1990), whereas the third pattern using periodic recurrence every 10 cycles (P_10_) resulted in a main frequency of 0.05 Hz (considering a respiratory rate 30 min^−1^) coinciding with dynamics of lung derecruitment and surfactant production and release (Bates and Irvin, 2002; Massa et al., 2008). All three variable *V*_*T*_ patterns were applied by remote control of the mechanical ventilator (Evita XL, Dräger Medical, Lübeck, Germany), as described by our group (Spieth et al., 2009b).

### Protocol for measurements

Measurements of gas exchange, hemodynamics, respiratory variables and distribution of ventilation were performed after instrumentation (Baseline 1) and induction of lung injury (Injury). Protective mechanical ventilation was then initiated in volume controlled ventilation mode with the following settings: *V*_*T*_ = 6 mL/kg, PEEP = 12 cm H_2_O, I:E = 1:1, RR ≤ 35 min^−1^ titrated to maintain pHa > 7.30 and FIO_2_ = 0.7. After a stabilization period of 30 min, measurements were repeated (Baseline 2). Following that, animals were randomly assigned to one of the following mechanical ventilation: (1) non-variable *V*_*T*_ (VCV); (2) variable *V*_*T*_ with white noise pattern (WN); (3) variable periodic *V*_*T*_ pattern with a periodic recurrence of 4 respiratory cycles (P_04_); and (4) variable periodic *V*_*T*_ pattern with a periodic recurrence of 10 respiratory cycles (P_10_). Other ventilator settings were kept constant in all groups, and animals ventilated during a period of 6 h, with measurements performed every hour (Times 1–6).

### Respiratory system mechanics

Airflow ($\overset{´}{V}$) was measured by the internal sensor of the mechanical ventilator. Airway pressure (*P*_*aw*_) was monitored by a pressure transducer (163PC01D48-PCB, Sensortechnics GmbH, Germany) placed at proximal side of the tracheal tube. Signals were acquired at sample frequency of 500 Hz using a data acquisition card (*NI USB-6210*, National Instruments, Austin, TX, USA) connected to a PC, and synchronized off-line according to the maximal co-variance criterion.

Respiratory system resistance (*R*) and elastance (*E* = *E*_1_+*E*_2_·*V*_*T*_) were determined by least-means-squares (LMS) fitting of the respiratory signals to the equation of motion:

$$\begin{array}{l} P_{aw}\left(t\right)=E_{1}\cdot V\left(t\right)+E_{2}\cdot V^{2}\left(t\right)+R\cdot \overset{´}{V}\left(t\right)+P_{0} \end{array}$$

where *E*_1_ is the volume-independent and *E*_2_ the volume-dependent *E*, and *P*_0_ the end-expiratory airway pressure. The contribution of volume-dependent *E* to total *E* (*%E*_2_) was calculated as described elsewhere (Kano et al., 1994).

The amount of inter-tidal recruitment-/derecruitment (R/D) was estimated from cycle-to-cycle differences in *E*. A given respiratory cycle was classified as -*slope* or +*slope* if its *V*_*T*_ was within a range of 6 mL/kg ± 0.045 mL/kg ($V_{T,i}^{+}$ and $V_{T,i}^{-}$) and its preceding cycle had a *V*_*T*_ value within 7–9 mL/kg ($V_{T,i-1}^{-}$) or 3-5 mL/kg ($V_{T,i-1}^{+}$*)*, respectively (Figure 1). The difference in *E* measured between −*slope* and +*slope* cycles was calculated as Δ*E* = *E*_−*slope*_ –* E*_+*slope*_. Negative Δ*E*-values were classified as inter-tidal recruitment, whereas positive Δ*E*-values were indicative of inter-tidal derecruitment.

### Distribution of regional ventilation

Regional ventilation distribution was assessed by electrical impedance tomography (EIT). Detailed methods and analysis of EIT are described in detail in Supplementary Material. Briefly, a 16 electrode belt was placed at mid chest circumference and connected to the EIT Evolution Kit 2 (Draeger Medical AG, Germany). The distribution of relative ventilation in ventral, central and dorsal regions was quantified, and the homogeneity, contrast and energy of tidal images were determined.

### Hemodynamics and gas exchange

Mean arterial and pulmonary arterial pressures were measured continuously (*MAP* and *MPAP*, respectively). Cardiac output (*CO*) was measured with a pulmonary artery catheter by thermodilution, and oxygen derived variables, including venous admixture, were obtained using standard formulae.

Arterial and mixed venous blood samples were analyzed for respiratory gases and pH using an ABL 505 blood gas analyzer (Radiometer, Copenhagen, Denmark), and for oxygen saturation and hemoglobin concentration with an OSM3 Hemoximeter (Radiometer) calibrated for swine blood. Gas tension measurements were performed at 37°C and corrected for body temperature measured by the pulmonary arterial catheter. To compensate for the influence of varying V_T_ on blood gases, blood samples were drawn along four to five respiratory cycles, 8–10 s respectively. During P_10_, two subsequent blood samples were taken and values averaged.

### Post mortem processing

At the end of the observation period, heparin was administered (1,000 IU/kg i.v.) (Ratiopharm, Ulm, Germany) and animals were killed by i.v. injection of 2 g thiopental (Inresa, Arzneimittel GmbH, Freiburg, Germany) followed by 50 mL KCl 1 M (Serumwerk Bernburg, Germany). Lungs were removed maintaining continuous positive airway pressure (*CPAP*) equal to the PEEP level during the observation period. Samples from gravitationally dependent (dorsal) and non-dependent (ventral) areas of the right lower lung lobe were snap-frozen in liquid nitrogen and stored at −80°C until further analysis. For tissue histologic evaluation, the left lower lung lobe was perfused with 4% buffered formaldehyde solution, while *CPAP* equivalent to the PEEP level during the observation period was maintained at the airway. Lung tissue samples of ~8 cm^3^ were taken from ventral and dorsal zones of the left lower lung lobe.

### Markers of inflammation and mechanical cell stress

Total RNA from lung tissue was isolated with TRI reagent (Sigma-Aldrich GmbH, Deisenhof, Germany) according to the manufacturer's protocol, followed by purification with NucleoSpin RNA II columns (Macherey&Nagel, Düren, Germany). The complementary deoxyribonucleic acid was synthesized with the Revert AidTM H Minus First Strand Synthesis Kit (MBI Fermentas, St. Leon Roth, Germany) from 1 μg total RNA according to instructions of the fabricant. Using cyclophilin A and ß2-microglobulin as housekeeping genes, the mRNA expression of the inflammatory mediators tumor necrosis factor α (TNF-α), interleukin 6 and 8 (IL-6, IL-8), amphiregulin and tenascin-c were quantified with quantitative real-time polymerase chain reaction (Maxima SYBR Green qPCR MasterMix, Fermentas, St. Leon Roth, Germany) in the iCycler MyiQ2 real time polymerase chain reaction system (BioRad, Munich, Germany). The total protein content in lung tissue was measured using the BioRad Protein Assay (BioRad, Munich, Germany). Protein levels of TNF-α, IL-6, and IL-8 were measured in lung tissue using commercial ELISA kits (R&D Systems, Wiesbaden, Germany) according to the manufacturer's instructions.

### Histological damage

Following perfusion fixation and immersion in 4% buffered formaldehyde solution for 7 days, tissue samples were embedded in paraffin, cut in slices of 5 μm thickness and stained with hematoxylin-eosin for further analysis. Photomicrographs at magnifications of x25, x100 and x400 were taken from four non-overlapping fields of view per section using a light microscope. Diffuse alveolar damage (*DAD*) was quantified by one of the authors (MK), who is an expert anatomist and was blinded to the therapy groups, using a weighted scoring system, as described elsewhere (Spieth et al., 2007). Briefly, values from 0 to 5 were used to represent the severity of seven features of *DAD*, i.e. alveolar edema, interstitial edema, hemorrhage, inflammatory infiltration, epithelial destruction, micro atelectasis and over distension, with 0 standing for no effect and 5 for maximum severity. Additionally, the extent of each feature characteristic per field of view was determined with values of 0 to 5, with 0 standing for no appearance and 5 for complete involvement. The cumulated DAD Score was calculated as the sum of a product of severity and extent of all features, resulting in values within the range from 0 to 175.

### Statistical analysis

The sample size calculations for testing the primary hypothesis (periodic *V*_*T*_ variation during variable ventilation improves PaO_2_/FIO_2_) was based on effects obtained from pilot studies. Accordingly, a sample size of 10 animals would provide appropriate power (1-β = 0.80) to identify a significant (α = 0.05) mean difference of at least 40 ± 60 mmHg, taking two-tailed tests and multiple comparisons (*n* = 6) into account (α^*^ = 0.0083, α^*^ Bonferroni adjusted).

Data are presented as mean ± standard deviation, unless stated otherwise. For lung function variables, group differences at Baseline 1, Injury and Baseline 2 were assessed using one-way ANOVA followed by pairwise *T*-test with *p*-value adjustment according to Bonferroni. Differences among groups were tested using two factorial repeated measures ANCOVA with between factor V_T_-pattern, within factor time and covariate of the respective parameter at Baseline 2. *Post-hoc* analysis and adjustment for multiple comparisons were performed according to Sidak's procedure. Lung histology, gene expression of pro-inflammatory markers as well as their protein levels in lung tissue were assessed using Kruskal–Wallis test followed by pairwise Mann–Whitney U test with adjustment for multiple comparisons according to Bonferroni–Holm procedure. Correlation analysis was performed using linear least mean squares modeling. A *p*-value of P ≤ 0.05 was considered statistically significant. Statistical analyses was performed using R Statistical programming language (R Core Team, 2016).

## Results

**Figure 2:**
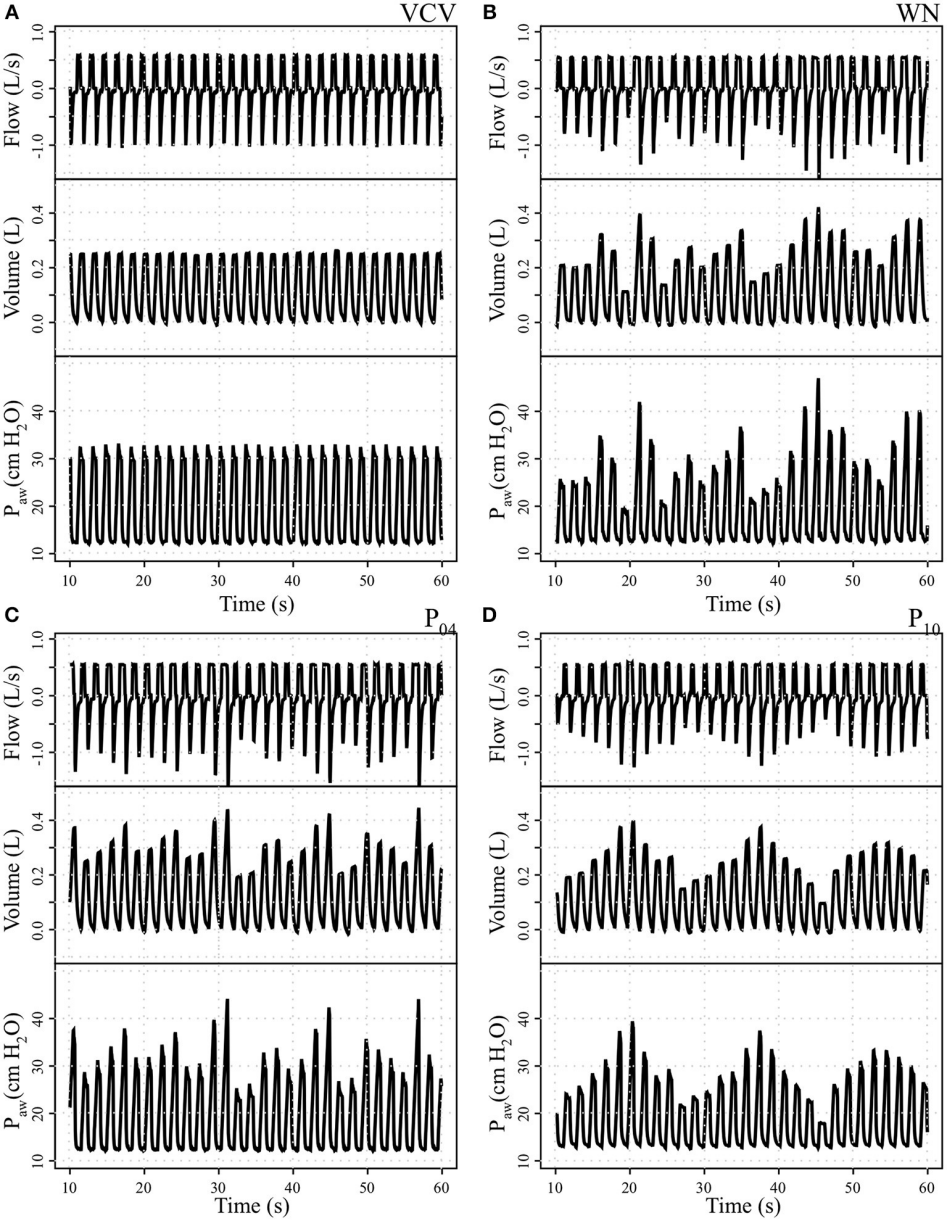
Tracing records of airway flow, volume, and airway pressure (P_aw_) in one representative animal per group. **(A)** Conventional volume controlled ventilation (VCV); **(B)** variable ventilation with Gaussian white noise pattern (WN); **(C)** variable ventilation with periodicity of 4 cycles (P_04_) and **(D)** variable ventilation with periodicity of 10 cycles (P_10_).

Bodyweight (38.2 ± 5.0, 42.1 ± 3.5, 42.4 ± 5.7, and 38.7 ± 6.9 kg in VCV, WN, P_04_ and P_10_, respectively) and number of lavages (11, 5–17; 10, 8–13; 10, 4–16, and 12, 5–19; median, min–max for VCV, WN, P_04_, and P_10_, respectively) did not differ among groups. Sample signal tracings at the start of therapy for each *V*_*T*_ pattern group are depicted in Figure 2.

**Figure 3:**
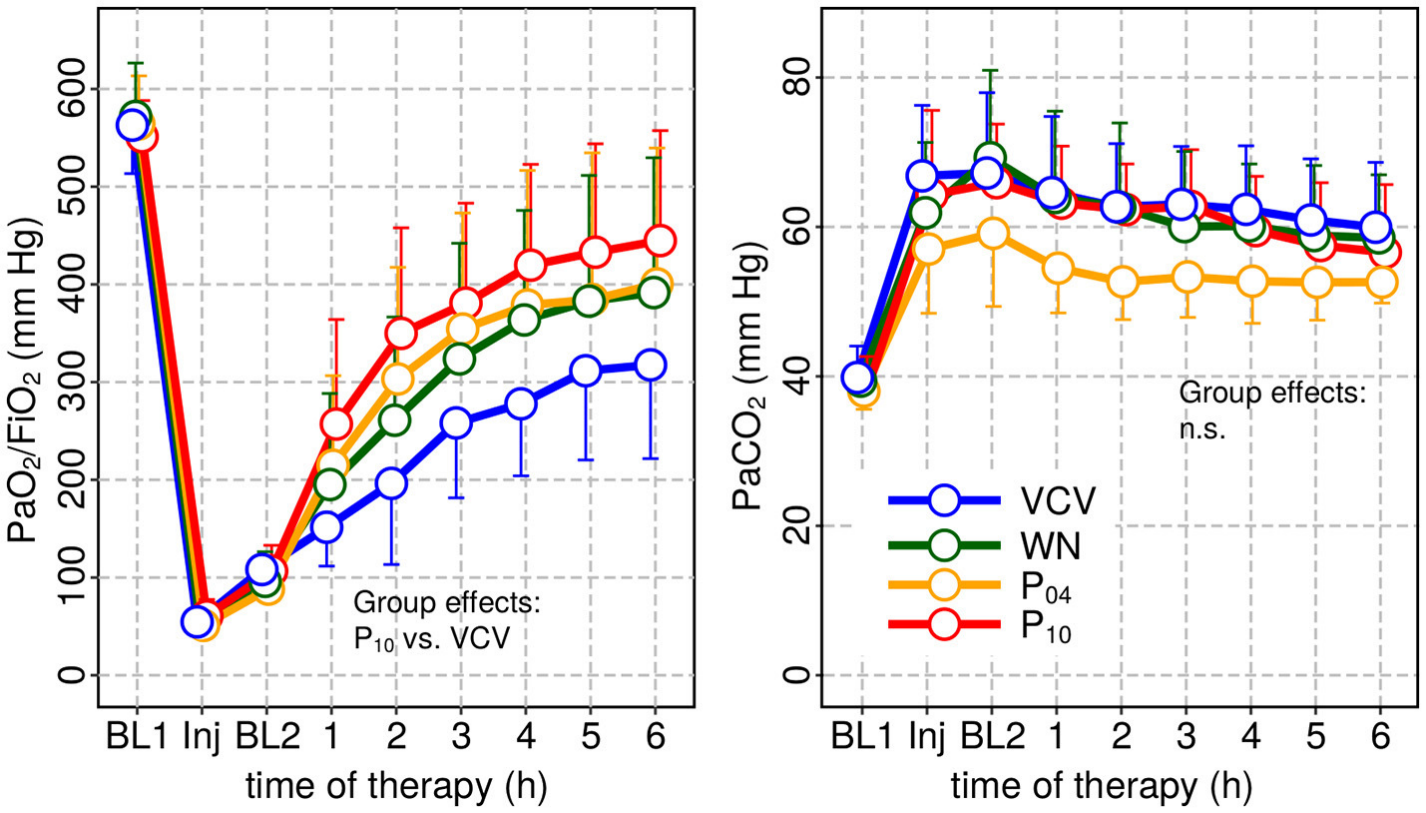
Arterial oxygenation (PaO_2_/F_I_O_2_) and arterial carbon-dioxide partial pressure PaCO_2_ at baseline 1 (BL1), injury and baseline 2 (BL2) and during subsequent therapy. PaO_2_ was significantly increased only for P_10_ compared to VCV.

Hemodynamics and gas exchange variables also did not differ among groups, except to PaO_2_/FIO_2_, which was higher in P_10_ compared to VCV (Figure 3 and Table 2).

**Table 2:** Hemodynamics and oxygen derived parameters.

**Variable**	**Group**	**BL1**	**Injury**	**BL2**	**Time 1**	**Time 2**	**Time 3**	**Time 4**	**Time 5**	**Time 6**	**Group effect**
${\overset{´}{\text{Q}}}_{\text{V}\text{A}}/{\overset{´}{\text{Q}}}_{\text{t}}$ **(%)**											
	VCV	11.8 ± 5.2	58.1 ± 09	44 ± 22	27.9 ± 15	20.1 ± 08	18 ± 7.5	25 ± 25	16 ± 5.7	15 ± 5.4	
	WN	10.8 ± 5.3	59.8 ± 16	45 ± 16	23.5 ± 12	24.5 ± 20	15 ± 5.7	13 ± 4.0	12 ± 4.7	12 ± 6.1	
	P_04_	11.9 ± 4.6	57.7 ± 13	44 ± 12	19.6 ± 6.2	14.5 ± 4.5	12 ± 3.5	12 ± 5.5	12 ± 6.1	12 ± 6.3	
	P_10_	13.0 ± 4.4	51.8 ± 15	37 ± 11	16.8 ± 3.6	14.0 ± 3.3	12 ± 2.7	11 ± 3.1	10 ± 5.0	09 ± 4.4	
**CO (L/min)**											
	VCV	4.6 ± 1	5.4 ± 2.1	4.9 ± 2.1	4.2 ± 1.4	3.7 ± 0.9	3.5 ± 0.7	3.6 ± 0.8	3.6 ± 0.8	3.5 ± 0.8	
	WN	5.0 ± 1.0	6.1 ± 1.6	5.1 ± 1.4	4.3 ± 0.7	3.9 ± 0.6	3.8 ± 0.6	3.9 ± 0.6	3.9 ± 0.5	3.9 ± 0.6	
	P_04_	5.3 ± 1.2	5.8 ± 1.7	5.3 ± 1.1	4.1 ± 1.1	3.7 ± 0.8	3.5 ± 0.6	3.5 ± 0.7	3.6 ± 0.8	3.5 ± 0.8	
	P_10_	4.7 ± 1.1	4.6 ± 0.7	4.4 ± 0.9	3.8 ± 0.8	3.6 ± 0.8	3.7 ± 0.7	3.4 ± 0.8	3.4 ± 0.8	3.4 ± 0.9	
**HR (min**^−1^**)**											
	VCV	97.5 ± 14	101 ± 14	96.0 ± 15	88.0 ± 12	83.2 ± 9	82.4 ± 10	79.1 ± 11	78.0 ± 10	79.5 ± 11.0	
	WN	94.1 ± 12	103 ± 15	93.4 ± 12	83.2 ± 12	82.1 ± 12	78.8 ± 10	77.0 ± 8	76.0 ± 6.6	73.2 ± 7.5	
	P_04_	92.8 ± 7.6	105 ± 19	98.1 ± 9.9	83.5 ± 7.8	80.7 ± 5.5	78.3 ± 3.4	75.7 ± 4.8	74.5 ± 5.9	72.2 ± 6.7	
	P_10_	101 ± 12	95.5 ± 15	92.0 ± 13	85.3 ± 10	83.6 ± 13	80.6 ± 14	77.3 ± 16	75.9 ± 16	76.8 ± 17	
**MAP (mm Hg)**											
	VCV	72 ± 11	89 ± 10	89 ± 14	90 ± 12	88 ± 10	86 ± 09	85 ± 09	82 ± 08	80 ± 09	
	WN	76 ± 09	89 ± 19	88 ± 11	87 ± 08	93 ± 11	87 ± 12	85 ± 12	81 ± 12	79 ± 09	
	P_04_	74 ± 13	92 ± 12	92 ± 11	91 ± 11	88 ± 10	88 ± 07	85 ± 10	83 ± 09	79 ± 07	
	P_10_	83 ± 13	94 ± 13	91 ± 13	89 ± 11	87 ± 09	86 ± 09	82 ± 10	79 ± 09	80 ± 13	
**MPAP (mm Hg)**											
	VCV	16.7 ± 1.6	32 ± 4.5.0	30.4 ± 3.7	29.2 ± 4.5	28.9 ± 4.1	31.0 ± 5.4	27.4 ± 4.3	27.0 ± 3.8	25.1 ± 4.0	
	WN	22.0 ± 19.0	33.9 ± 5.0	30.7 ± 5.5	28.3 ± 4.3	27.9 ± 3.7	28.1 ± 2.9	28.1 ± 2.6	27.0 ± 2.7	26.4 ± 2.7	
	P_04_	17.4 ± 4.6	32.2 ± 5.9	31.7 ± 4.9	26.7 ± 4.0	27.2 ± 3.9	27.4 ± 4.2	27.3 ± 4.6	27.2 ± 4.2	26.3 ± 3.8	
	P_10_	19.3 ± 3.3	33.6 ± 3.5	30.3 ± 4.3	27.6 ± 4.7	27.1 ± 5.2	27.0 ± 4.8	25.1 ± 4.5	24.3 ± 4.8	25.0 ± 4.5	

*Ratio of partial arterial oxygen pressure to fraction of inspired oxygen (P/F Ratio); intrapulmonary shunt ($\frac{{\overset{´}{Q}}_{VA}}{{\overset{´}{Q}}_{t}}$); partial arterial pressure of carbon dioxide (PaCO_2_); cardiac output (CO); heart rate (HR); mean arterial blood pressure (MABP); mean pulmonary arterial blood pressure (MPAP); conventional volume controlled ventilation (VCV); variable volume controlled ventilation with Gaussian white noise pattern in tidal volume (WN); variable volume controlled ventilation with periodicity of 4 cycles (P_04_) and variable volume controlled ventilation with periodicity of 10 cycles (P_10_). Values are shown as mean and standard deviation, and were obtained from 40 animals in total (n = 10/group). There were no missing values. Statistical significance was accepted at P < 0.05. Comparability of groups at Injury and BL2 was tested using one-way ANOVA followed by Bonferroni post-hoc tests. Differences among groups (group main effect) were tested with general linear model statistics using values at BL2 as covariate and adjusted for repeated measurements according to the Sidak procedure*.

**Figure 4:**
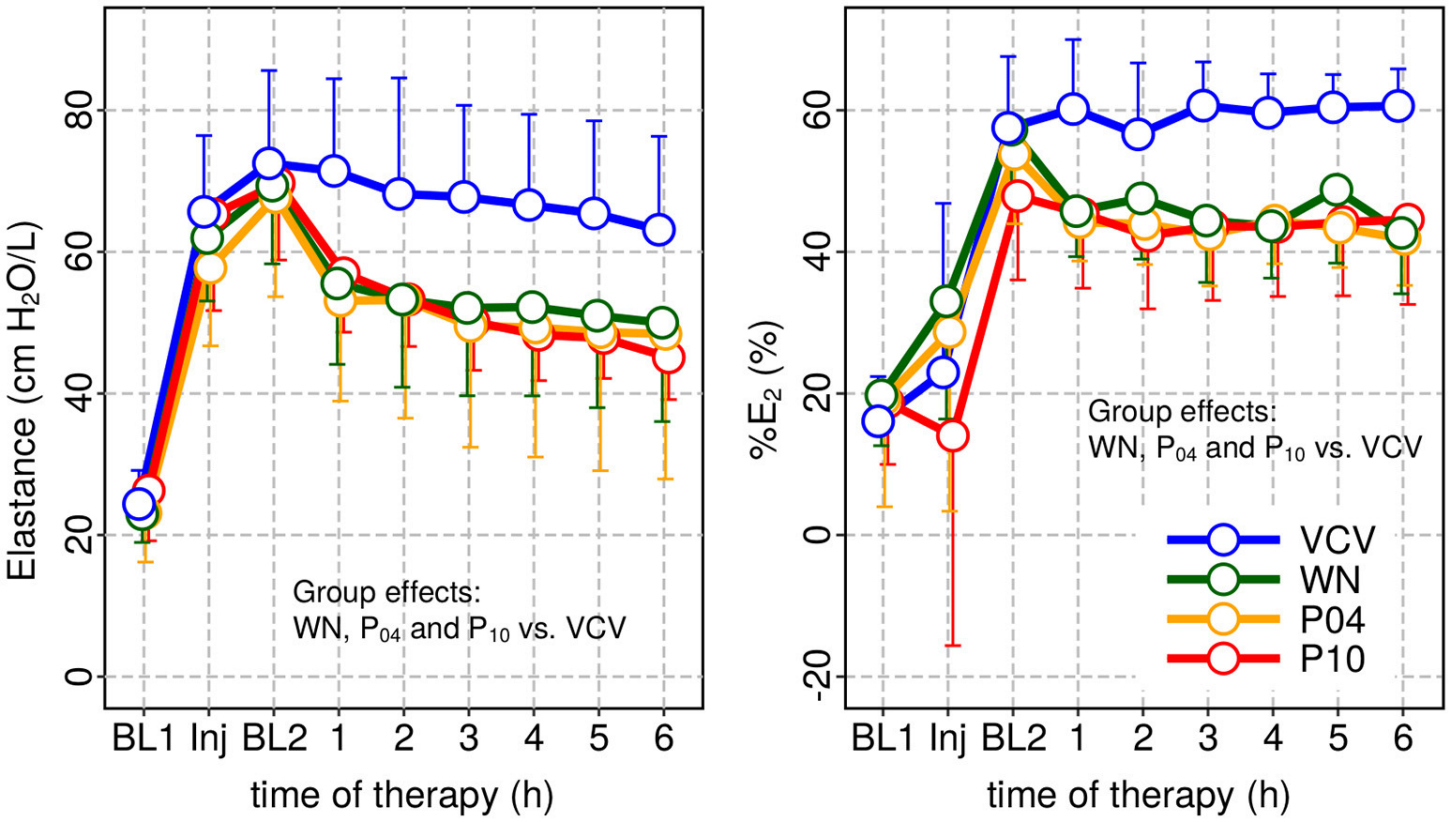
Dynamic respiratory system elastance (*E*) and relative volume dependence of *E (%E*_2_) at baseline 1 (BL1), injury and baseline 2 and during subsequent therapy period. In all patterns of variable ventilation *E* and *%E*_2_ were significantly reduced compared to VCV.

**Figure 5:**
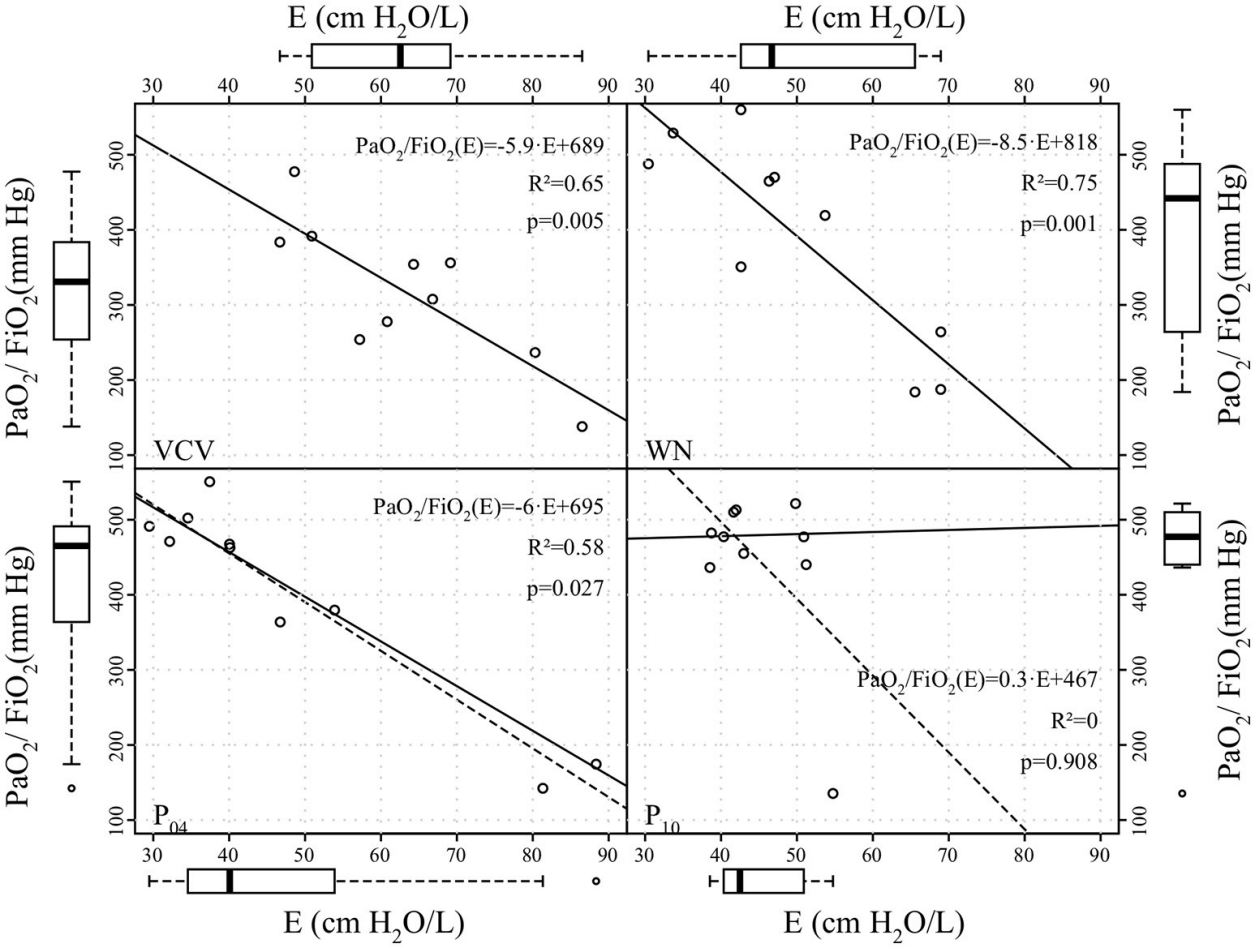
Correlation between arterial partial oxygen pressure to inspiratory oxygen fraction ratio (PaO_2_/FiO_2_) and dynamic respiratory system elastance (*E*) at end of the therapy period. Straight continuous and dashed lines represent linear regression lines without and with outliers, respectively; the coefficients of determination (*R*^2^) were calculated excluding outliers.

Peak airway pressure *P*_*aw, peak*_, plateau airway pressure *P*_*aw, plat*_ and mean airway pressure *P*_*aw, mean*_ were significantly reduced in WN, P_04_ and P_10_ compared to VCV. *E, E*_2_, and *%E*_2_ were reduced in WN, P_04_ and P_10_ compared to VCV, whereas *R* did not differ among groups (Table 3 and Figure 4). In VCV, WN and P_04_, but not P_10_, correlation analyses revealed that PaO_2_/FIO_2_ decreased proportionally to *E* (Figure 5).

**Table 3:** Respiratory variables.

**Parameter**	**Group**	**BL1**	**Injury**	**BL2**	**Time 1**	**Time 2**	**Time 3**	**Time 4**	**Time 5**	**Time 6**	**Group effect**
MV (L·min^−1^)	VCV	5.2 ± 0.9	5.6 ± 1.0	8.2 ± 1.3	8.1 ± 1.2	8.0 ± 1.1	7.6 ± 0.7	7.5 ± 0.7	7.5 ± 0.7	7.5 ± 0.7	
	WN	5.8 ± 0.9	6.9 ± 1.5	9.1 ± 1.2	8.9 ± 1.1	8.9 ± 1.2	8.7 ± 1.2	8.5 ± 1.1	8.3 ± 1.1	8.2 ± 1.2	
	P_04_	5.8 ± 1.2	6.1 ± 1.4	9.1 ± 1.3	8.9 ± 1.3	8.4 ± 1.5	8.3 ± 1.4	8.1 ± 1.3	7.9 ± 1.1	7.5 ± 1.0	
	P_10_	5.4 ± 1.0	5.9 ± 1.4	8.6 ± 1.6	8.1 ± 1.2	8.1 ± 1.3	7.9 ± 1.5	7.9 ± 1.5	7.5 ± 1.6	7.6 ± 1.7	
V_T_ (mL/kg)	VCV	9.9 ± 0.2	10.8 ± 0.4	6.6 ± 0.1	6.6 ± 0.1	6.6 ± 0.2	6.6 ± 0.1	6.6 ± 0.1	6.6 ± 0.1	6.6 ± 0.1	
	WN	9.9 ± 0.7	10.3 ± 1.2	6.5 ± 0.2	6.4 ± 0.2	6.4 ± 0.2	6.4 ± 0.2	6.4 ± 0.2	6.4 ± 0.1	6.4 ± 0.1	
	P_04_	10.3 ± 0.6	10.5 ± 0.7	6.6 ± 0.1	6.5 ± 0.2	6.3 ± 0.4	6.4 ± 0.1	6.4 ± 0.2	6.5 ± 0.1	6.4 ± 0.1	
	P_10_	10.0 ± 0.4	10.7 ± 0.4	6.6 ± 0.2	6.3 ± 0.4	6.3 ± 0.4	6.3 ± 0.4	6.4 ± 0.4	6.3 ± 0.6	6.4 ± 0.4	
RR (min^−1^)	VCV	13.7 ± 2.2	13.6 ± 2.2	32.3 ± 3.4	32.3 ± 2.5	31.8 ± 2.8	30.5 ± 4.0	30.1 ± 3.9	30.2 ± 4.3	30.2 ± 4.3	
	WN	13.9 ± 1.9	16.4 ± 5.9	33.1 ± 3.6	33.2 ± 3.5	33.0 ± 4.2	32.1 ± 4.1	31.5 ± 3.9	30.9 ± 3.7	30.4 ± 3.8	
	P_04_	13.4 ± 2.3	13.6 ± 2.4	32.5 ± 1.9	32.5 ± 2.4	31.5 ± 3.5	30.4 ± 3.6	29.9 ± 3.5	29.0 ± 3.4	27.8 ± 3.8	
	P_10_	14.3 ± 2.8	14.6 ± 3.3	33.8 ± 1.8	33.8 ± 1.7	33.5 ± 1.6	32.3 ± 2.4	32.3 ± 2.4	31.2 ± 2.1	31.9 ± 3.3	
P_aw_, _peak_ (cm H_2_O)	VCV	18.2 ± 1.6	36.9 ± 4.5	32.4 ± 2.3	32.0 ± 2.2	31.1 ± 2.4	30.8 ± 1.9	30.5 ± 1.9	30.2 ± 1.9	29.7 ± 2.0	*P* < 0.001
	WN	18.5 ± 1.6	37.3 ± 4.1	34.0 ± 3.3	30.2 ± 3.2	29.4 ± 3.4	29.1 ± 3.4	29.1 ± 3.3	28.7 ± 3.6	28.5 ± 4.0	**
	P_04_	19.3 ± 2.7	36.4 ± 3.7	33.3 ± 2.7	29.5 ± 3.4	28.9 ± 3.8	28.3 ± 3.8	28.1 ± 3.9	27.9 ± 4.3	27.6 ± 4.4	**
	P_10_	18.8 ± 1.7	37.7 ± 4.6	32.2 ± 3.1	28.8 ± 3.5	28.0 ± 3.4	27.3 ± 3.5	26.9 ± 3.5	26.9 ± 3.4	26.0 ± 3.5	**
P_aw_, _plat_ (cm H_2_O)	VCV	14.9 ± 1.2	30.5 ± 1.9	29.8 ± 2.2	29.5 ± 2.0	28.8 ± 2.3	28.6 ± 1.9	28.3 ± 1.9	28.1 ± 2.0	27.7 ± 2.1	*P* < 0.001
	WN	15.4 ± 1.2	32.5 ± 2.9	31.4 ± 3.1	27.9 ± 3.3	27.3 ± 3.5	27.0 ± 3.5	27.0 ± 3.5	26.6 ± 3.7	26.4 ± 4.1	**
	P_04_	15.4 ± 3.3	30.8 ± 3.2	30.9 ± 2.8	27.3 ± 3.2	26.8 ± 3.6	26.1 ± 3.6	25.9 ± 3.6	25.7 ± 4.1	25.4 ± 4.3	***
	P_10_	15.1 ± 1.6	31.0 ± 3.4	29.4 ± 2.4	26.5 ± 3.1	25.7 ± 3.1	25.1 ± 3.1	24.7 ± 3.1	24.2 ± 3.1	23.8 ± 3.2	***
P_aw_, _mean_ (cm H_2_O)	VCV	10.4 ± 0.5	17.4 ± 1.2	20.0 ± 0.8	19.9 ± 0.8	19.5 ± 0.9	19.5 ± 0.7	19.4 ± 0.7	19.3 ± 0.7	19.2 ± 0.7	*P* < 0.001
	WN	10.4 ± 0.6	18.5 ± 1.8	20.6 ± 1.2	18.9 ± 1.2	18.6 ± 1.3	18.5 ± 1.3	18.5 ± 1.3	18.3 ± 1.4	18.4 ± 1.5	***
	P_04_	10.5 ± 1.1	17.1 ± 1.5	20.1 ± 1.0	18.5 ± 1.1	18.5 ± 1.3	18.1 ± 1.2	18.1 ± 1.3	17.8 ± 1.5	17.9 ± 1.5	***
	P_10_	10.4 ± 0.8	17.1 ± 1.7	19.9 ± 1.2	18.4 ± 1.3	18.1 ± 1.2	17.8 ± 1.3	17.6 ± 1.3	17.8 ± 1.1	17.3 ± 1.2	***
R (cm H_2_O/L/s)	VCV	6.6 ± 1.0	11.5 ± 3.1	6.9 ± 1.1	6.7 ± 1.0	6.3 ± 0.9	6.2 ± 0.9	6.2 ± 0.9	6.1 ± 0.9	5.9 ± 0.9	
	WN	6.5 ± 1.7	10.8 ± 2.9	7.0 ± 1.7	6.3 ± 1.1	5.8 ± 1.0	5.8 ± 1.0	5.8 ± 1.0	5.6 ± 1.2	5.6 ± 1.0	
	P_04_	6.8 ± 1.1	10.6 ± 2.2	6.7 ± 1.0	6.2 ± 1.0	5.9 ± 1.2	5.9 ± 1.0	5.6 ± 1.1	5.6 ± 1.1	5.5 ± 1.1	
	P_10_	7.4 ± 1.3	12.1 ± 2.9	7.4 ± 1.4	6.6 ± 1.4	6.3 ± 1.3	6.1 ± 1.2	5.9 ± 1.2	6.1 ± 0.9	5.8 ± 1.2	
E_1_ (cm H_2_O/L)	VCV	20.5 ± 4.1	50.5 ± 21	33.0 ± 8.3	30.8 ± 8.2	28.5 ± 7.0	26.2 ± 6.0	26.0 ± 6.0	25.6 ± 5.5	24.7 ± 6.0	
	WN	17.2 ± 3.1	45.1 ± 13	30.6 ± 6.7	30.1 ± 7.1	28.5 ± 8.1	27.3 ± 9.0	28.1 ± 8.7	26.0 ± 9.2	26.3 ± 8.2	
	P_04_	18.9 ± 3.7	44.4 ± 20	30.4 ± 9.0	29.1 ± 7.8	29.0 ± 10.0	27.7 ± 9.4	26.9 ± 10.0	26.1 ± 9.9	26.0 ± 11.0	
	P_10_	21.6 ± 5.0	51.9 ± 22	32.8 ± 8.8	31.6 ± 7.6	29.2 ± 6.6	27.0 ± 6.7	25.9 ± 6.1	26.7 ± 5.6	25.2 ± 6.3	
E_2_ (cm H_2_O/L^2^)	VCV	12 ± 6.0	39 ± 44	156 ± 53	162 ± 52	160 ± 65	166 ± 52	162 ± 50	159 ± 49	154 ± 48	*P* < 0.001
	WN	11 ± 1.9	44 ± 31	141 ± 40	95 ± 27	93 ± 31	90 ± 27	87 ± 25	90 ± 28	85 ± 33	***
	P_04_	10 ± 12	24 ± 33	139 ± 50	93 ± 39	87 ± 43	82 ± 46	85 ± 49	84 ± 53	83 ± 54	***
	P_10_	13 ± 8.4	39 ± 69	152 ± 67	108 ± 31	97 ± 31	93 ± 29	89 ± 28	86 ± 27	77 ± 28	***

Baseline 1 (BL1), Injury (IN), Baseline 2 (BL2). Minute ventilation (MV); respiratory rate (RR); mean V_T_ per kg (V_T_); peak airway pressures (P_aw_, _peak_); plateau airway pressures (P_aw_, _plat_); mean airway pressure (P_aw_, _mean_); resistance of the respiratory system (R); elastance of the respiratory system (E); volume-independent elastance (E_1_); volume dependent elastance (E_2_); volume-dependence index of respiratory system elastance (%E_2_); conventional volume controlled ventilation (VCV); variable volume controlled ventilation with Gaussian white noise pattern in tidal volume (WN); variable volume controlled ventilation with periodicity of 4 cycles (P_04_) and variable volume controlled ventilation with periodicity of 10 cycles (P_10_). Values are shown as mean and standard deviation, and were obtained from 40 animals in total (n = 10/group). There were no missing values. Statistical significance was accepted at p < 0.05. Comparability of groups at Injury and BL2 was tested using one-way ANOVA followed by Bonferroni post-hoc tests. Differences among groups (group main effect) were tested with general linear model statistics using values at BL2 as covariate and adjusted for repeated measurements according to the Sidak procedure

(** p < 0.01,

*** *p < 0.001)*.

**Figure 6:**
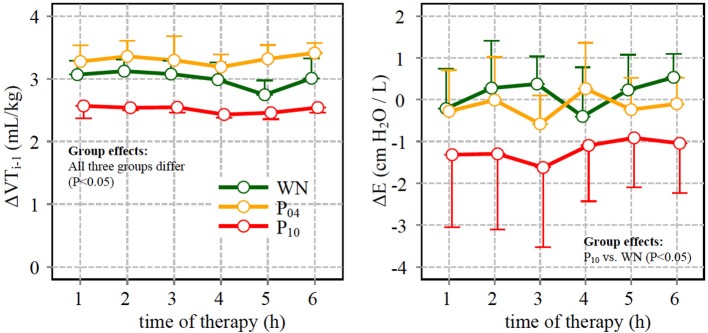
**Left:** difference between tidal volume (*V*_*T*_) in the descending limb of a given cycle and in the ascending limb of the preceding cycle (Δ*V*_*T*,i−1_) (see also text and Figure 1); **Right**: difference of dynamic respiratory system elastance (ΔE) calculated for cycles with V_T_ value close to the mean (6 mL/kg) in the descending and in the ascending slopes (*V*_*T*_i__- and *V*_*T*_i__+, respectively) of the respective pattern.

Compared to WN, Δ*E* was significantly higher within the P_10_ pattern, although the tidal volume of the respective preceding cycle Δ*V*_*T, i*−1_ was lowest in the P_10_ group (Figure 6).

The distribution of regional ventilation did not differ significantly among groups. Analysis of tidal EIT images showed that homogeneity and energy increased, whereas contrast decreased during variable ventilation compared to VCV, irrespective of the *V*_*T*_ pattern (see Supplementary Material).

**Figure 7:**
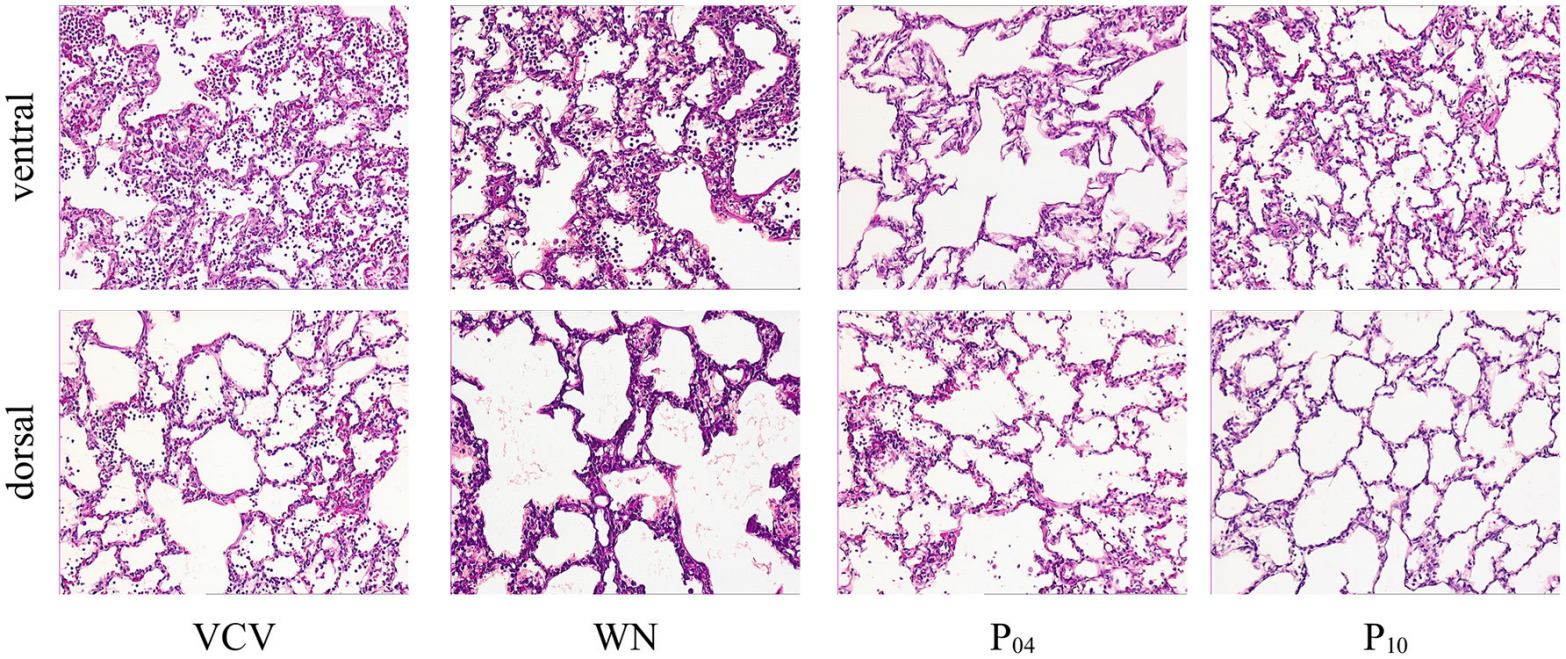
Histological slides with HE-staining at 100-fold magnification of representative animals.

DAD Score (Table 4 and Figure 7), gene expression of markers of inflammation and cell mechanical stress (Table 5), and protein content of pro-inflammatory markers in lung tissue (Table 6), did not differ significantly among groups.

**Table 4:** Diffuse alveolar damage.

**Feature**	**group**	**Ventral region**	**group effect**	**Dorsal region**	**Group effect**
Alveolar edema	VCV	2[0–4]	n.s.	1[0–2]	n.s.
	WN	4[1–6]		1[0–4]	
	P_04_	1[0–8]		2[0–4]	
	P_10_	2[0–6]		1[1–3]	
Intersitial edema	VCV	1[1–4]	n.s.	1[0–4]	n.s.
	WN	2[1–4]		1[1–3]	
	P_04_	1[1–4]		1[1–2]	
	P_10_	2[1–4]		1[1–4]	
Hemorrhage	VCV	1[0–3]	n.s.	1[0–1]	n.s.
	WN	2[1–4]		1[0–2]	
	P_04_	1[0–4]		2[0–3]	
	P_10_	2[0–6]		1[0–3]	
Inflammatory infiltration	VCV	8[3–12]	n.s.	3[1–6]	n.s.
	WN	12[5–15]		3[2–6]	
	P_04_	6[2–12]		5[2–8]	
	P_10_	8[1–12]		3[1–8]	
Epithelial destruction	VCV	4[1–4]	n.s.	1[0–4]	n.s.
	WN	3[1–6]		1[1–6]	
	P_04_	1[0–6]		2[1–9]	
	P_10_	1[1–8]		4[1–9]	
Microatelectasis	VCV	4[1–4]	n.s.	1[1–4]	n.s.
	WN	2[1–4]		1[1–2]	
	P_04_	2[1–4]		2[1–4]	
	P_10_	2[1–4}		1[1–2]	
Overdistension	VCV	3[1–6]	n.s.	1[0–3]	n.s.
	WN	4[1–8]		1[0–2]	
	P_04_	5[0–8]		2[0–3]	
	P_10_	6[4–7]		1[0–3]	
Cumulative score	VCV	26[13–33]	n.s.	12[3–20]	n.s.
	WN	33[18–43]		13[7–17]	
	P_04_	21[5–48]		18[8–27]	
	P_10_	26[9–45]		14[6–25]	

*Diffuse alveolar damage in ventral and dorsal lung regions. Conventional volume controlled ventilation (VCV); variable volume controlled ventilation with Gaussian white noise pattern (WN); variable volume controlled ventilation with periodicity of 4 cycles (P_04_) and variable volume controlled ventilation with periodicity of 10 cycles (P_10_). Values are shown as median and interquartile range, and were obtained from 40 animals in total (n = 10/group). There were no missing values. Differences among groups were tested with Kruskal-Wallis test followed by Mann-Whitney U-test and Bonferroni-Holm adjustments for pairwise comparisons. n.s (non-significant at P < 0.05)*.

**Table 5:** Gene expression of markers of inflammation and cell stress.

**Marker**	**Group**	**Ventral region**	**Group effect**	**Dorsal region**	**Group effect**
TNF-α	VCV	1.0	n.s.	1.0	n.s.
	WN	1.0 [0.6–1.9]		0.7 [0.3–1.0]	
	P_04_	1.0 [0.7–1.7]		0.6 [0.5–1.5]	
	P_10_	1.4 [0.7–2.3]		0.7 [0.5–1.3]	
IL-6	VCV	1.0	n.s.	1.0	n.s.
	WN	0.9 [0.6–1.2]		0.9 [0.5–1.5]	
	P_04_	0.9 [0.2–1.3]		0.7 [0.4–1.3]	
	P_10_	0.8 [0.3–1.4]		0.8 [0.4–1.4]	
IL-8	VCV	1.0	n.s.	1.0	n.s.
	WN	1.5 [0.8–2.1]		1.2 [0.5–2.1]	
	P_04_	1.4 [0.2–3.6]		0.3 [0.2–2.5]	
	P_10_	1.5 [0.4–2.7]		0.9 [0.6–1.3]	
Amphiregulin	VCV	1.0	n.s.	1.0	n.s.
	WN	0.9[0.5–1.6]		1.2 [0.5–2.2]	
	P_04_	1.2 [0.5–2.0]		0.5 [0.3–1.4]	
	P_10_	1.6 [0.6–3.5]		1.0 [0.7–1.5]	
Tenascin-c	VCV	1.0	n.s.	1.0	n.s.
	WN	1.3 [0.6–2.2]		1.0 [0.5–2.2]	
	P_04_	1.2 [0.8–2.1]		1.2 [0.8–1.8]	
	P_10_	1.0 [0.6–5.6]		1.6 [0.5–2.2]	

*Gene expression of Tumor necrosis factor (TNF)-α, Interleukin (IL)-6, Interleukin (IL)-8, Amphiregulin and Tenascin-c in ventral lung regions (ventral) and dorsal lung regions (dorsal), conventional volume controlled ventilation (VCV); variable volume controlled ventilation with Gaussian white noise pattern in tidal volume (WN); variable volume controlled ventilation with periodicity of 4 cycles (P_04_) and variable volume controlled ventilation with periodicity of 10 cycles (P_10_). Values represent x-fold expression of the respective gene normalized to housekeeping genes cyclophilin A and ß2-microglobulin and to values of VCV animals as control group. Values are shown as median and interquartile range, and were obtained from 40 animals in total (n = 10/group). There were no missing values. Differences among groups were tested with Kruskal-Wallis test followed by Mann-Whitney U-test and Bonferroni-Holm adjustments for pairwise comparisons. n.s. (non-significant at P < 0.05)*.

**Table 6:** Levels of inflammation markers in lung tissue.

**Marker**	**Group**	**Ventral region**	**Group effect**	**Dorsal region**	**Group effect**
TNF-α (pg/mg)	VCV	2.8 [1.9–7.5]	n.s.	3.0 [2.4–5.8]	n.s.
	WN	2.4 [0.4–4.7]		4.3 [1.4–10.7]	
	P_04_	3.2 [1.1–4.8]		3.8 [1.0–5.6]	
	P_10_	1.1 [0.6–4.8]		2.9 [1.1–7.4]	
IL-6 (pg/mg)	VCV	39.5 [18.9–102.8]	n.s.	28.2 [8.6–50.1]	n.s.
	WN	31.8 [18.7–50.0]		34.9 [13.7–106.3]	
	P_04_	26.0 [14.4–59.2]		14.4 [7.0–44.8]	
	P_10_	39.1 [23.4–60.5]		20.4 [8.4–34.9]	
IL-8 (pg/mg)	VCV	133.7 [63.1–327.5]	n.s.	106.1 [62.5–286.2]	n.s.
	WN	118.0 [48.1–226.0]		125.0 [68.8–216.4]	
	P_04_	120.3 [37.5–474.1]		88.75 [48.5–208.0]	
	P_10_	90.6 [70.5–118.0]		101.9 [72.5–130.9]	

*Gene expression of Tumor necrosis factor (TNF)-α, Interleukin (IL)-6, Interleukin (IL)-8, in ventral lung regions (ventral) and dorsal lung regions (dorsal), conventional volume controlled ventilation (VCV); variable volume controlled ventilation with Gaussian white noise pattern in tidal volume (WN); variable volume controlled ventilation with periodicity of 4 cycles (P_04_) and variable volume controlled ventilation with periodicity of 10 cycles (P_10_). Values are shown as median and interquartile range, and were obtained from 40 animals in total (n = 10/group). There were no missing values. However, a variable number of measurements yielded values below the detection limit of the respective ELISA kit and were excluded from the analysis. The remaining numbers of values were: in ventral regions for TNF-α VCV 9, WN 8, P_04_ 8, P_10_ 10 animals; for IL-6 VCV 10, WN 10, P_04_ 9, P_10_ 10 animals; for IL-8 VCV 10, WN 10, P_04_ 10, P_10_ 9 animals; in dorsal regions for TNF-α VCV 9, WN 8, P_04_ 8, P_10_ 10 animals; for IL-6 VCV 8, WN 8, P_04_ 9, P_10_ 10 animals; for IL-8 VCV 10, WN 10, P_04_ 10, P_10_ 9 animals. Differences among groups were tested with Kruskal-Wallis test followed by Mann-Whitney U-test and Bonferroni-Holm adjustment for pairwise comparison. n.s. (non-significant at P < 0.05)*.

## Discussion

The main finding of this study was that, in an experimental model of severe ARDS in pigs, periodic *V*_*T*_ fluctuation at a frequency of 0.05 Hz enhanced oxygenation and increased inter-tidal R/D during variable ventilation.

To our knowledge, this is the first study comparing different types of variable ventilation with periodic V_T_ fluctuation in a large animal model of severe ARDS. Three tidal volume patterns with distinct amplitude-frequency spectra were compared to conventional controlled mechanical ventilation. While WN has, by definition, a constant, frequency independent spectrum amplitude, there were distinct peaks in the spectra of the pattern P_04_ and P_10_ at one quarter (f_P04_ = 0.13 Hz) and one tenth (f_P10_ = 0.05 Hz) of the average respiratory frequency (0.5 Hz), respectively. In mice with hydrochloric acid injured lungs, a decaying probability density distribution tailored toward high *V*_*T*_ values outperformed the original variable ventilation pattern based on a Gaussian distribution with respect to lung mechanics and gas exchange (Thammanomai et al., 2008, 2013), likely due to lung recruitment (Graham et al., 2011b). Different from these previous studies, we selected *V*_*T*_ patterns with identical probability density distributions.

Our data showed that oxygenation was significantly improved only in P_10_, when *V*_*T*_ fluctuated at a frequency of 0.05 Hz. In contrast, *E* was significantly reduced in all variable ventilation groups compared to VCV, a finding that is in line with previous studies (Arold et al., 2002; Funk et al., 2004; Spieth et al., 2009b). The correlation analysis revealed that, with increasing *V*_*T*_ fluctuation period, variations in PaO_2_/FiO_2_ were less well explained by variations in *E*. There are two possible explanations for this observation: (1) with increasing period lung recruitment was more stable over multiple respiratory cycles, for example due to enhanced surfactant production/release; (2) periodic *V*_*T*_ fluctuation redistributed perfusion more effectively, improving ventilation/perfusion matching.

To investigate whether inter-tidal R/D cycling occurs along with periodic *V*_*T*_ variation we performed the cycle-type analysis selecting respiratory cycles with mean *V*_*T*_ whose preceding cycle had a higher or lower *V*_*T*_, −slope or +slope. Due to the selection criteria that were chosen in order to gain a comparable number of cycles independent of the *V*_*T*_ pattern and cycle class, the difference of *V*_*T*_ of the preceding cycles Δ *V*_*T*,i+1_ differed between *V*_*T*_ patterns being significantly lower in P_10_ compared to P_04_ and WN. Therefore, Δ*E* should be of lesser magnitude for P_10_ compared to the other patterns, if inter-tidal R/D would be absent. However while Δ*E* was not significantly different from zero in patterns WN and P04, it was significantly negative in P_10_ (Figure 6), indicating the presence of inter-tidal R/D of two or more cycles in P_10_. It is worth noting that this type of analysis would yield differences between −slope and +slope cycles in every viscoelastic tissue or material with relevant hysteresis independent of R/D. However the increased difference between cycle types is more likely due to a type of prolonged R/D dynamics in agreement with (Bellardine et al., 2006), as P_10_ proved to be the only type of variable ventilation able to improve oxygenation. This may suggest that R/D on a shorter time scale, which may occur during ventilation with reduced or no periodicity in tidal volume patterns, is less effective in improving lung function in this type of injury.

The reduction of the frequency *V*_*T*_ pattern amplified arterial oxygenation *PaO*_2_. The possible mechanism may be stabilization of lung recruitment by increased inter-tidal but decreased intra-tidal R/D. But other factors such as surfactant production/release and redistribution of perfusion may be triggered by periodic *V*_*T*_ variation and may contribute additionally to improvements in lung function. However arterial oxygenation may not increase during an indefinite reduction of *V*_*T*_ pattern frequency, since a *V*_*T*_ frequency of zero corresponds to conventional volume controlled ventilation. If the *V*_*T*_ frequency of maximal arterial oxygenation coincides with the frequency response of one participating subsystem of respiration, this frequency dependent response would be characteristic for deterministic resonance. The identification of such frequency and related subsystem were beyond the scope of this investigation. However further research on this issue would not only foster our knowledge of the respiratory system and its interactions but would facilitate further development of protective ventilation strategies.

Several mechanisms may explain the improvement of gas exchange and lung mechanics through periodic *V*_*T*_ patterns. Lung recruitment can be facilitated by surfactant release due to stretch of lung epithelial type II cells. Its magnitude is related to an instantaneous Ca^2+^ mobilization decaying with a time constant of 9.13 s (Wirtz and Dobbs, 1990) and simultaneous surfactant secretion with a time constant of 19.7 s (Haller et al., 1998) to 94.6 s (Majumdar et al., 2012). Therefore, in lung areas subjected to inter-tidal R/D high *V*_*T*_ cycles may activate surfactant secretion more effectively, while this surfactant is released after the strain impulse has decayed during subsequent low *V*_*T*_ cycles (Edwards, 2001).

In contrast to intra-tidal, inter-tidal R/D may have beneficial effects on ventilation/perfusion matching due to redistribution of perfusion toward lung regions with improved ventilation (Sylvester et al., 2012). The transient response of local acute hypoxic pulmonary (HPV) vasoconstriction is characterized by a time constant of 151 ± 24.8 s in humans (Morrell et al., 1995) and 120 s in dogs (Grant and Schneider, 1983). Thus, only 1.6% of the final HPV response is reached during intra-tidal R/D (approximately T = 2 s), whereas 8% is reached in regions that remain open/closed for ~10 s, which was likely achieved in P_10_.

The dynamics of formation and resolution of atelectasis differ according to the type of lung injury, as well as PEEP levels. In a lung lavage model in pigs, duration of atelectasis formation varied from 20.0 ± 12.3 to 26.5 ± 14.0 s, while duration of atelectasis resolution ranged from 0.8 ± 0.6 to 10.7 ± 4.2 s (Neumann et al., 1998; Markstaller et al., 2001). This likely reduced cyclic R/D, explaining in part the benefits of the lower main frequency during variable ventilation. Possibly, a prolongation of the *V*_*T*_ period up to a T_P_ = 40 s (f_P_ = 0.025 Hz) might yield a further increase on oxygenation, but this claim remains speculative.

The effects of periodicity of *V*_*T*_ patterns on gas exchange and lung mechanics have been investigated in one experimental and one simulation study. In pigs with oleic acid-induced lung injury, biologically and white noise, computer-generated variable ventilation led to comparable lung mechanics and gas exchange (Froehlich et al., 2008). However, the so-called Hurst exponent H, i.e. a measure of long range correlation, might not have differed relevantly between both patterns (H = 0.62 for biological noise and H = 0.5 for computer generated white noise) considering its spectrum of possible values for H = 0 anti-correlation (so-called blue noise), H = 0.5 (no correlation, white noise, respiratory rate during deep sleep) and H = 1 auto-correlation (so-called pink noise, respiratory rate during awake state; Schumann et al., 2010).

In the presented study the novelty was that we introduced a deterministic auto-correlation with a fixed frequency similar to the numerical simulation study by Ma et al. In their model, taking R/D dynamics into account, increased periodicity did not improve lung mechanics. However, different from *in-vivo* models, other possible factors affected by periodicity, for example surfactant release and redistribution of perfusion and ventilation were not considered in their model (Ma et al., 2011).

Although changes in the distribution of tidal ventilation did not reach statistical significance, increased homogeneity in tidal impedance images in all variability groups suggests increased lung homogeneity during variable ventilation. However, in order to avoid artifacts of the moving diaphragm, a rather cranial, cross section of the lung was addressed, and we cannot exclude the possibility that R/D occurred in the most dependent (dorsal and caudal) lung regions, contributing to the observed effects of variable ventilation on lung function.

No relevant differences among groups were found regarding lung histology, gene expression as well as protein levels of markers of inflammation in lung tissue, which is somewhat in disagreement with previous work from our own group (Spieth et al., 2009b). A possible explanation is that in the present study we used a model of severe ARDS, which might be less responsive to variable ventilation.

The improvement of oxygenation through periodic variable ventilation ΔPaO_2_/F_I_O_2_ = 150 mmHg, seen in this study, compares well to other techniques as for example random variable ventilation ΔPaO_2_/F_I_O_2_ = 80 … 150 mm Hg (Huhle et al., 2016) and step-wise recruitment maneuvers' ΔPaO_2_/F_I_O_2_ = 150 mm Hg(Vivona et al., 2018) in the same model. In the double-hit model facilitated in this study the effects of recruitment on oxygenation might still be over-estimated compared to clinical ARDS where recruitment maneuvers' only improve oxygenation by ΔPaO_2_/F_I_O_2_ = 40 … 50 mm Hg (Hodgson et al., 2011). Furthermore the full benefits of periodic variable ventilation and the relevant mechanism remain to be elucidated.

Periodic variable ventilation is straightforward implementable in experimental and conventional ventilators that are remotely controllable as e.g., the Inspira ASVp (Harvard Apparatus, Massachusetts, USA), the FlexiVent (SciReq, Montreal, Canada) and Evita XL (Draeger Medical, Luebeck, Germany).

## Limitations

Our study has several limitations. First, the applied experimental model of ARDS consisted of repeated surfactant lavage (first hit) followed by consecutive ventilator induced lung injury (second hit). This model of ARDS reproduces important features of human ARDS (Matute-Bello et al., 2008), but it does not reflect the complex clinical picture of ARDS precluding direct extrapolation of our results to the clinical scenario. Second, the observational period was limited to 6 h, and we cannot exclude different effects in the long term. Third, to enhance comparability between groups, we kept settings for FIO_2_ and PEEP constant during the observational period avoiding adjustments based on changes in gas exchange. A clinical approach including such adaptations may have produced different results. Fourth, a further prolongation of the *V*_*T*_ period may have yielded different effects of periodicity on lung function in this animal model, but were not investigated. Last but not least the Gaussian distribution of the *V*_*T*_ patterns used might not be tailored to the species and model of ARDS.

## Conclusions

In this experimental model of severe ARDS, periodic *V*_*T*_ fluctuation at a frequency of 0.05 Hz improved oxygenation during variable ventilation, suggesting that deterministic resonance adds further benefit to variable ventilation.

## Data availability statement

The datasets used and/or analyzed during the current study are available from the corresponding author on reasonable request.

## Author contributions

AG and RH contributed to design the study, conducted experiments, analyzed, and interpreted all experimental data, and were major contributors in writing the manuscript. AB contributed to design the study, analyzed and interpreted data on respiratory mechanics, and contributed in writing the manuscript. TK and TB conducted experiments, analyzed and interpreted all experimental data, and were major contributors in writing the manuscript. IR contributed to design the study, analyzed, and interpreted data on molecular biology, and contributed in writing the manuscript. SK and NC contributed to design the study, analyze all data and write the manuscript. MK contributed to design the study, to analyze data on histologic damage, and contributed in writing the manuscript. PP contributed to design the study, analyzed and interpreted all experimental data, and was a major contributor in writing the manuscript. MA designed the study, obtained financial support, analyzed and interpreted all experimental data, and was a major contributor in writing the manuscript. All authors read and approved the final manuscript.

### Conflict of interest statement

MA has been granted patents on variable pressure support ventilation, which were licensed to Dräger Medical AG, Lübeck, Germany. The remaining authors declare that the research was conducted in the absence of any commercial or financial relationships that could be construed as a potential conflict of interest.

## Abbreviations

ARDS: Acute respiratory distress syndrome
CPAP: Continuous positive airway pressure ventilation mode
CV: Coefficient of variation in %, defined as CV =^*^100 with V_T,I_
CVP: Central venous pressure in mm Hg
DAD: Diffuse alveolar damage score
*E*: Total respiratory system elastance: E = E_1_ + E_2_ · V_T_ in cm H_2_Oml^−1^
E_1_: Volume independend respiratory system elastance in cm H_2_O·ml^−1^
E_2_: Volume dependend respiratory system elastance in cm H_2_O·ml^−2^
%E_2_: Volume dependence index of respiratory system elastance in %
HR: Heart rate in min^−1^
MAP: Mean arterial pressure in mm Hg
MPAP: Mean pulmonary arterial pressure in mm Hg
P_0_: Minimal airway pressure per respiratory cycle in cm H_2_O
WN, P_04_, P_10_: Pattern of variable ventilation without, every 4 and every 10 resp. cycles recurrence (periodicity)
P_aw, mean|plat|max_: Minimal, mean, plateau and maximal airway pressure in cm H_2_O
PCWP: Pulmonary capillary wedge pressure in mm Hg
PEEP: Positive end-expiratory pressure in cm H_2_O
R: Respiratory system resistance in cm H_2_O·s·ml^−1^
R/D: Recruitment / derecruitment: repeated opening and closing of lung regions
RR: Respiratory rate set at the ventilator in min^−1^
VCV: Conventional volume controlled ventilation
*V*_*T*_: Mean inspiratory tidal volume in ml·kg^−1^ actual body weight
*V*_*T, I*_: Inspiratory tidal volume of the i-th respiratory cycle.
