# Supplementary material for: Periodic Fluctuation of Tidal Volumes Further Improves Variable Ventilation in Experimental Acute Respiratory Distress Syndrome
Source: Front Physiol. 2018 Jul 12;9:905. doi: 10.3389/fphys.2018.00905 (PMC6052143; doi:10.3389/fphys.2018.00905)
Supplement: Supplementary file 1 [file Data_Sheet_1.docx]

Supplementary Material

Periodic fluctuation of tidal volumes further improves variable ventilation in experimental acute respiratory distress syndrome

Andreas Güldner^1#^, Robert Huhle^1#^, Alessandro Beda^1,2^, Thomas Kiss^1^, Thomas Bluth^1^, Ines Rentzsch^1^, Sarah Kerber^1^, Nadja C. Carvalho^1,2^, Michael Kasper^3^, Paolo Pelosi^4^, Marcelo Gama de Abreu^1*^

*** Correspondence:** Marcelo Gama de Abreu: mgabreu@ukdd.de

# Stochastic resonance, Deterministic resonance and Variable Ventilation

Stochastic resonance in a term coined in physics to describe a phenomenon of a non-linear system (as the respiratory system) that may improve its output performance (gas exchange and lung mechanics) when its input (tidal volume) varying or uncertain when compared to constant input (Figure 1).


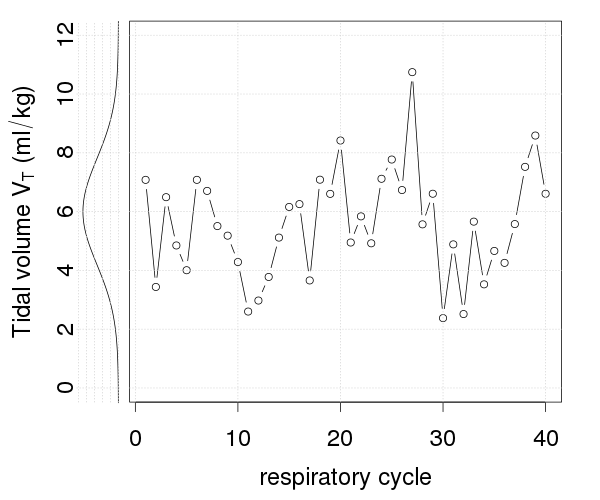


**Respiratory System**

PaO_2_ ↗

Resp. Elastance ↘

**STOCHASTIC RESONANCE**

Supplementary Figure 1 – Pattern and distribution of tidal volume during random variable white noise ventilation and its beneficial effects on the output (gas exchange, PaO_2_) and state (elastance) of the respiratory system.

In physiological spontaneous breathing tidal volume is not constant but varies, depending on the physiological state by about 20 to 30 % of its mean. The primary hypothesis for variable ventilation thus was to simulate this physiological variation during controlled mechanical ventilation. The main identified mechanism of variable ventilation is the recruitment of collapsed lung areas through intermitted high tidal volumes that leads to an improvement in gas exchange and lung mechanics.

However the application of completely random physiological variability to a pathological system may not maximize positive effects. E.g. biological subsystems involved with respiration as hypoxic pulmonary vasoconstriction or surfactant production and release do show distinct inertia related to the underlying biochemical processes to adjust to the recruitment state of the lung. The function and interaction of those subsystems might be improved when using patterns that show a distinct periodicity.

# Advanced signal properties of tidal volume patterns

The time course, power spectral density and the characteristic properties of the tidal volume patterns are shown in Supplementary Figure 2 and Supplementary Table 1. While all variability patterns had the same values for mean and coefficient of variation of *V_T_*, the complexity expressed by distribution independent sample entropy (Richman and Moorman, 2000) was lowest in P_10_ and highest in WN. Furthermore P_04_ and P_10_ had distinct power spectral density distribution with maxima at 0.13 Hz and 0.05 Hz, respectively, at a mean respiratory rate of 30 min^-1^.


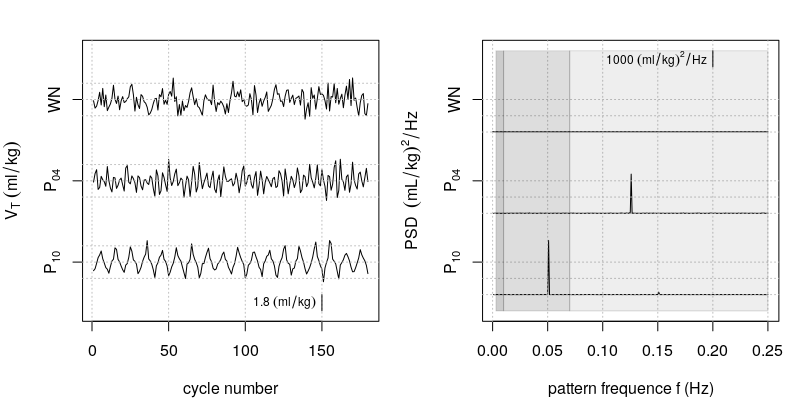


Supplementary Figure 2 – *V_T_* patterns in time and in frequency domain.

Supplementary Table 1 – Characteristic properties of the patterns used for (variable) volume controlled ventilation.

|  | WN | P_04_ | P_10_ |
| --- | --- | --- | --- |
| Mean *V_T_* (mL∙kg^-1^) | 6 | 6 | 6 |
| Coefficient of variation (%) | 30 | 30 | 30 |
| *V_T_* Periodicity / Cycle duration (sec) | none | 7.7 | 20 |
| *V_T_* Frequency (Hz) | none | 0.13 | 0.05 |
| Peak PSD, normalized (none) | none | 0.31 | 0.43 |
| Sample Entropy (none) | 2.2 | 1.7 | 0.9 |

*V_T_* frequency is the frequency at global maximum in the power spectral density distribution (PSD) denoted by Peak PSD; signal to noise ratio quantifies the ratio between the periodic (best-fit sine function) and the random fluctuations; sample entropy, a distribution independent measure of complexity (>2 for uncorrelated noise and 0 for a pure sine wave). For *V_T_* Periodicity / Cycle duration, *V_T_* Frequency and Peak PSD a mean respiratory rate of 30 bpm is assumed.

# Electronic impedance tomography analysis

Eletrical impedance tomographic measurement was performed with the Draeger EIT Evolution Kit 2 (Draeger Medical AG, Germany) with a sixteen electrode belt placed at mid chest circumference. Measurements were done with a frame rate of 20 Hz and the stimulation current had amplitude of 8 µA and a frequency of 100 kHz.

The 208 raw voltage values per frame were used for 64x64 conductance image reconstruction using EIDORS (Adler and Lionheart, 2006). The cross sectional thorax shape largely differs from the human cross sectional thorax shape (Grychtol et al., 2012) and thus the earlier was constructed from end-expiratory computer tomographic images from a previous study (Carvalho et al., 2011). Creation of a custom FEM model of a pig thorax used for solving the inverse problem was done according to the GREIT approach (Adler et al., 2009). Lung region of interest was determined for each experiment using linear regression analysis of local per pixel impedance changes to global impedance changes as described in (Pulletz et al., 2006).

The region of interest (ROI) was segmented into three regions with equal height along the dorsal-ventral axis (EIT_ventral_, EIT_central_, and EIT_dorsal_) and the relative tidal ventilation measured. Additionally the homogeneity, contrast and energy of tidal images were assessed using the grey-level co-occurrence approach described elsewhere (Haralick et al., 1973).

The distribution of regional ventilation did not differ significantly among V_T_ patterns of variability, but P_No_ led to an increase of tidal ventilation in EIT_dors_ regions, as compared to VCV (Supplemental Figure 1). Analysis of tidal images showed that homogeneity and energy increased, whereas contrast decreased during variable ventilation compared to control, irrespective of the V_T_ pattern (Supplemental Table 1).

Supplemental Figure 1 – Change of tidal ventilation compared to baseline in the dorsal ROI and change of tidal image homogeneity as obtained using the EIT.

**Supplementary Table 2 – Distribution of tidal ventilation and Homogeneity and Complexity of Tidal EIT**

| Parameter | Group | BL2 | Time 1 | Time 2 | Time 3 | Time 4 | Time 5 | Time 6 | group effect |
| --- | --- | --- | --- | --- | --- | --- | --- | --- | --- |
| ventral (%) | VCV | 0 ± 0 | 0 ± 1 | 0 ± 1 | 0 ± 1 | 0 ± 2 | 0.2 ± 1 | 1.4 ± 2 | n.s. |
|  | WN | 0 ± 0 | -2 ± 2 | -2 ± 2 | -2 ± 2 | -1 ± 3 | -1.5 ± 1 | -1.1 ± 2 |  |
|  | P_04_ | 0 ± 0 | -1 ± 2 | -2 ± 2 | -2 ± 2 | -1 ± 2 | -1.9 ± 2 | -1.5 ± 2 |  |
|  | P_10_ | 0 ± 0 | -2 ± 2 | -2 ± 3 | -2 ± 3 | 0 ± 4 | 0.1 ± 3 | -0.5 ± 4 |  |
|  |  |  |  |  |  |  |  |  |  |
| mid-ventral (%) | VCV | 0 ± 0 | -1 ± 2 | -2 ± 1 | -3 ± 1 | -3 ± 1 | -2.7 ± 2 | -3.2 ± 2 | n.s. |
|  | WN | 0 ± 0 | -4 ± 2 | -4 ± 2 | -4 ± 2 | -6 ± 3 | -6.2 ± 2 | -6.1 ± 2 |  |
|  | P_04_ | 0 ± 0 | -2 ± 1 | -4 ± 1 | -4 ± 2 | -4 ± 2 | -4.9 ± 3 | -5.1 ± 3 |  |
|  | P_10_ | 0 ± 0 | -2 ± 2 | -3 ± 2 | -4 ± 2 | -5 ± 3 | -5.0 ± 3 | -5.1 ± 3 |  |
|  |  |  |  |  |  |  |  |  |  |
| mid-dorsal (%) | VCV | 0 ± 0 | -1 ± 1 | -1 ± 2 | -1 ± 1 | 0 ± 4 | -1.4 ± 1 | -2.6 ± 1 | n.s. |
|  | WN | 0 ± 0 | -1 ± 2 | -1 ± 3 | -1 ± 3 | -2 ± 2 | -2.2 ± 2 | -2.4 ± 2 |  |
|  | P_04_ | 0 ± 0 | -1 ± 5 | 0 ± 1 | 0 ± 2 | -1 ± 3 | -1.3 ± 2 | -1.5 ± 3 |  |
|  | P_10_ | 0 ± 0 | -1 ± 2 | 0 ± 2 | 0 ± 2 | -1 ± 2 | -1.7 ± 4 | -1.2 ± 3 |  |
|  |  |  |  |  |  |  |  |  |  |
| dorsal (%) | VCV | 0 ± 0 | 2 ± 2 | 3 ± 2 | 4 ± 2 | 4 ± 2 | 4.1 ± 3 | 4.5 ± 3 | P < 0.05 |
|  | WN | 0 ± 0 | 6 ± 3 | 7 ± 3 | 7 ± 3 | 8 ± 3 | 8.7 ± 4 | 9.3 ± 3 | *** |
|  | P_04_ | 0 ± 0 | 5 ± 2 | 6 ± 2 | 7 ± 3 | 7 ± 2 | 7.8 ± 5 | 7.9 ± 4 |  |
|  | P_10_ | 0 ± 0 | 4 ± 4 | 6 ± 3 | 6 ± 4 | 7 ± 4 | 6.9 ± 6 | 7.3 ± 5 |  |
|  |  |  |  |  |  |  |  |  |  |
| Contrast C | VCV | 250±50 | 250±30 | 250±50 | 230±50 | 210±70 | 230±50 | 210±60 | P < 0.001 |
| (none) | WN | 250±40 | 88±30 | 85±30 | 83±10 | 91±20 | 100±20 | 89±20 | *** |
|  | P_04_ | 240±80 | 84±20 | 91±20 | 93±20 | 100±20 | 98±30 | 100±30 | *** |
|  | P_10_ | 230±30 | 110±20 | 92±20 | 90±40 | 110±20 | 100±20 | 100±20 | *** |
|  |  |  |  |  |  |  |  |  |  |
| Energy E  (none) | VCV | 1.2±0.1 | 1.2±0.09 | 1.2±0.09 | 1.2±0.09 | 1.2±0.2 | 1.2±0.1 | 1.2±0.1 | P < 0.001 |
|  | WN | 1.1±0.2 | 1.6±0.4 | 1.8±0.8 | 1.6±0.3 | 1.6±0.4 | 1.6±0.3 | 1.5±0.3 | *** |
|  | P_04_ | 1.2±0.1 | 1.7±0.4 | 1.6±0.2 | 1.6±0.1 | 1.6±0.1 | 1.6±0.2 | 1.5±0.2 | ** |
|  | P_10_ | 1.1±0.1 | 1.4±0.1 | 1.4±0.2 | 1.7±0.9 | 1.4±0.1 | 1.4±0.2 | 1.4±0.1 | * |
|  |  |  |  |  |  |  |  |  |  |
| Homogeneity H | VCV | 0.16±0.01 | 0.16±0.02 | 0.16±0.01 | 0.17±0.02 | 0.18±0.04 | 0.17±0.02 | 0.17±0.02 | P < 0.001 |
| (none) | WN | 0.16±0.02 | 0.24±0.04 | 0.25±0.05 | 0.24±0.02 | 0.24±0.03 | 0.23±0.02 | 0.24±0.01 | *** |
|  | P_04_ | 0.17±0.03 | 0.25±0.04 | 0.23±0.02 | 0.23±0.02 | 0.23±0.02 | 0.24±0.03 | 0.23±0.02 | *** |
|  | P_10_ | 0.15±0.02 | 0.21±0.02 | 0.22±0.02 | 0.24±0.07 | 0.22±0.02 | 0.23±0.03 | 0.23±0.02 | *** |
|  |  |  |  |  |  |  |  |  |  |

Change of tidal ventilation and homogeneity of tidal impedance images measured by electrical impedance tomography: ventral, mid-ventral, mid-dorsal and dorsal regions of the global lung region of interest (ROI); conventional volume controlled ventilation (VCV); variable volume controlled ventilation with Gaussian white noise V_T_ pattern (WN); variable volume controlled ventilation with periodicity of 4 cycles (P_04_) and variable volume controlled ventilation with periodicity of 10 cycles (P_10_). Values are shown as mean and standard deviation. Due to technical problems with the EIT device, values were obtained from 38 animals in total, n=10/group except of P_10_. Comparability of groups at Injury and BL2 was tested using one-way ANOVA followed by Bonferroni post-hoc tests. Differences among groups (group main effect) were tested with general linear model statistics using values at BL2 as covariate and adjusted for repeated measurements according to the Sidak procedure (post-hoc effect: * vs. VCV). Statistical significance was accepted at P < 0.05.

# References

Adler, A., Arnold, J. H., Bayford, R., Borsic, A., Brown, B., Dixon, P., et al. (2009). GREIT: a unified approach to 2D linear EIT reconstruction of lung images. *Physiol. Meas.* 30, S35. doi:10.1088/0967-3334/30/6/S03.

Adler, A., and Lionheart, W. R. B. (2006). Uses and abuses of EIDORS: an extensible software base for EIT. *Physiol. Meas.* 27, S25. doi:10.1088/0967-3334/27/5/S03.

Carvalho, A. R., Spieth, P. M., Güldner, A., Cuevas, M., Carvalho, N. C., Beda, A., et al. (2011). Distribution of regional lung aeration and perfusion during conventional and noisy pressure support ventilation in experimental lung injury. *J. Appl. Physiol. Bethesda Md 1985* 110, 1083–1092. doi:10.1152/japplphysiol.00804.2010.

Grychtol, B., Lionheart, W. R. B., Bodenstein, M., Wolf, G. K., and Adler, A. (2012). Impact of model shape mismatch on reconstruction quality in electrical impedance tomography. *IEEE Trans. Med. Imaging* 31, 1754–1760. doi:10.1109/TMI.2012.2200904.

Haralick, R. M., Shanmugam, K., and Dinstein, I. (1973). Textural Features for Image Classification. *Syst. Man Cybern. IEEE Trans. On* 3, 610–621. doi:10.1109/tsmc.1973.4309314.

Pulletz, S., Genderingen, H. R. van, Schmitz, G., Zick, G., Schädler, D., Scholz, J., et al. (2006). Comparison of different methods to define regions of interest for evaluation of regional lung ventilation by EIT. *Physiol. Meas.* 27, S115. doi:10.1088/0967-3334/27/5/S10.

Richman, J. S., and Moorman, J. R. (2000). Physiological time-series analysis using approximate entropy and sample entropy. *Am. J. Physiol. - Heart Circ. Physiol.* 278, H2039–H2049. Available at: http://ajpheart.physiology.org/content/278/6/H2039 [Accessed April 2, 2013].
